# Presenilin1 regulates Th1 and Th17 effector responses but is not required for experimental autoimmune encephalomyelitis

**DOI:** 10.1371/journal.pone.0200752

**Published:** 2018-08-08

**Authors:** Matthew Cummings, Anitha Christy Sigamani Arumanayagam, Picheng Zhao, Sunil Kannanganat, Olaf Stuve, Nitin J. Karandikar, Todd N. Eagar

**Affiliations:** 1 Department of Pathology, University of Texas Southwestern Medical Center, Dallas, TX, United States of America; 2 Department of Pathology and Genomic Medicine, Houston Methodist Hospital Research Institute, Houston, TX, United States of America; 3 Neurology Section, VA North Texas Health Care System, Medical Service, Dallas, TX, United States of America; 4 Department of Pathology, University of Iowa, Iowa City, IA, United States of America; University of Texas at San Antonio, UNITED STATES

## Abstract

Multiple Sclerosis (MS) is an inflammatory demyelinating disease of the central nervous system (CNS) where pathology is thought to be regulated by autoreactive T cells of the Th1 and Th17 phenotype. In this study we sought to understand the functions of Presenilin 1 (PSEN1) in regulating T cell effector responses in the experimental autoimmune encephalomyelitis (EAE) murine model of MS. PSEN1 is the catalytic subunit of γ-secretase a multimolecular protease that mediates intramembranous proteolysis. γ-secretase is known to regulate several pathways of immune importance. Here we examine the effects of disrupting PSEN1 functions on EAE and T effector differentiation using small molecule inhibitors of γ-secretase (GSI) and T cell-specific conditional knockout mice (PSEN1 cKO). Surprisingly, blocking PSEN1 function by GSI treatment or PSEN1 cKO had little effect on the development or course of MOG35-55-induced EAE. *In vivo* GSI administration reduced the number of myelin antigen-specific T cells and suppressed Th1 and Th17 differentiation following immunization. *In vitro*, GSI treatment inhibited Th1 differentiation in neutral but not IL-12 polarizing conditions. Th17 differentiation was also suppressed by the presence of GSI in all conditions and GSI-treated Th17 T cells failed to induce EAE following adoptive transfer. PSEN cKO T cells showed reduced Th1 and Th17 differentiation. We conclude that γ-secretase and PSEN1-dependent signals are involved in T effector responses *in vivo* and potently regulate T effector differentiation *in vitro*, however, they are dispensable for EAE.

## Introduction

Multiple sclerosis (MS) is an inflammatory, demyelinating and neurodegenerative disorder of the central nervous system (CNS). Pathology in MS is associated with inflammatory lesions, demyelination, axonal disruption and neuronal loss in the white and grey matter of the CNS. It is thought that myelin-specific autoimmune T cells are important contributors to the CNS pathology (reviewed in [1, 2]). MS has been associated with increases in myelin-reactive T cells (reviewed in [3]). In fact, T cells with a Th1 or Th17 phenotypes have been associated with disease [4–8]. The essential role of T cells in MS is supported by work using the experimental autoimmune encephalomyelitis (EAE) animal model. EAE has been extensively utilized to study the functions of T cells in regulating inflammation in the CNS. The generation of disability in EAE is associated with myelin-reactive T cells that produce IFNγ, IL-17 and GM-CSF [9–14]. Conversely, T cells with a regulatory (Treg) phenotype are thought to negatively impact disease and promote homeostasis in EAE [15, 16]. Thus, there is an effort to define pathways that modify the generation of pathogenic T effector cells while promoting the function of anti-inflammatory Treg cells.

Presenilin 1 (PSEN1) is a multi-pass transmembrane protein expressed by many immune cell types. PSEN1 is best known for its role as the catalytic subunit of γ-secretase, a large multi-molecular protein complex composed of four core components: Nicastrin, Presenilin 1, anterior pharynx-defective 1(Aph1) and presenilin enhancer 2 (Pen2). The γ-secretase complex is known to be involved in the degradation and removal of proteins from the membrane. γ-secretase has been found to act on a large number of substrates including several pathways of immune importance; cadherin proteins, CD44, interleukin-1 receptor (IL-1R1), MHC class I, TGFβ Receptor (TGFβR1) and Notch receptors (reviewed in [17]). In addition to its functions in γ-secretase, PSEN1 has also been described to regulate signaling pathways independent of its proteolytic activity. Evidence from developmental models suggests that PSEN1 plays a role in regulating calcium, β-catenin and cell death independent of γ-secretase [18].

Pharmacological inhibitors of γ-secretase have been developed and tested in preclinical models and clinical trials for Alzheimer’s disease (reviewed in [19, 20]). Several of these small molecule inhibitors have been applied in models of inflammation [21–29]. GSI treatment was also found to reduce the severity of EAE by regulating effector T cell function [30–32], altering macrophage activity [33] or by promoting remyelination [34, 35].

Given the potential for γ-secretase to regulate pathways involved in cell migration, Th1 and Th17 differentiation and antigen presentation, we sought to understand the contributions of PSEN1 and γ-secretase in autoimmune T cell responses using the MOG35-55 model of EAE. Treatment with GSI had minimal effects on the course of EAE despite reducing Th1 and Th17 T cell numbers in the CNS. *In vivo* administration of GSI was found to reduce the numbers of myelin-specific T cells and suppress Th1 and Th17 differentiation following immunization. Mechanistic studies demonstrated that PSEN1 regulated Th1 differentiation as measured by IFNγ, Tbet and IL12Rb2 expression. Similarly, Th17 differentiation was inhibited with reduced expression of IL-17, RORγt, IL12Rb1 and IL23R. GSI was also associated with altered CD25 expression and reduced T cell proliferation *in vitro*. The effects of GSI administration on Th1 differentiation could be overcome by the addition of IL-2 and IL-12. To test the T cell-intrinsic role of γ-secretase, experiments were performed using T cell-specific PSEN1 conditional knockout mice (PSEN1 cKO). PSEN1 cKO mice had a weak reduction in EAE severity. Th1 and Th17 effector T cells were found at normal frequencies following immunization. *In vitro* experiments with T cells from PSEN1 cKO donors showed defects in Th1 and Th17 differentiation with reduced proliferation. We conclude that PSEN1 and γ-secretase are not essential for MOG35-55-induced EAE. The data support a model where PSEN1-dependent signals influence T cell responses at the level of T cell proliferation, Th1 and Th17 differentiation but are not required for pathogenic T cell responses.

## Materials and methods

### Mice

Naïve mice were purchased or bred in the laboratory. 8–10 week old female C57Bl/6 mice were purchased from Taconic. CD4-Cre transgenic mice [36], PSEN1 lox/lox mice [37], 2D2 TCR transgenic mice [38] and CD90.1 congenic mice were purchased from Jackson. Animal experiments were approved by the IACUC at HMHRI or UTSW. B10.PL/J mice were purchased from Jackson Laboratories. MBP 1–11 TCR transgenic mice [39] were bred at UTSW. All animals were housed under SPF conditions.

### EAE induction

Active EAE was induced in C57/BL.6 mice by subcutaneous immunization of 200μl of complete Freund’s adjuvant (CFA) (Difco) containing 30μg of MOG35-55, as described [40]. On days 0 and 2, each mouse was injected with 200ng pertussis toxin (Toxin Technologies). Adoptive EAE was induced by the transfer of 5x10^6^ MBP1-11 TCR transgenic T cells that had been *in vitro* polarized to a Th1 or Th17 effector phenotype as indicated. EAE severity was scored following a 5-point scale as previously described [41]. Experiments were repeated at least once.

### Inhibitors

Dibenzazepine (DBZ) was purchased from Cayman. *In vivo*, mice received intraperitoneal injections every other day consisting of 100μl of a solution of GSI (0.1mg) dissolved in DMSO and olive oil or vehicle alone (DMSO with olive oil). Mice were randomized into treatment groups. DMSO and GSI-treated animals were co-housed. *In vitro*, GSI was dissolved in DMSO and was added to the culture media at a final concentration of 0.01μM. Control cells were incubated with an equal concentration of DMSO. Cells were treated with GSI or DMSO for 30 minutes prior to stimulation.

### Antibodies, peptides and recombinant proteins

Synthetic MOG35-55 was purchased from Anaspec. MBP ac1-11 was purchased from Genemed. Recombinant cytokines used *in vitro* include rhIL-2 at 10u/ml (Peprotech), rIL-12 at 10ng/ml (Biolegend). The following antibodies were utilized in cell culture, all were purchased from BioXcell: anti-CD3 (145-2C11), anti-CD28 (PV-1) and anti-IL-4 (clone 11B11). The following fluorophore-conjugated antibodies were used for flow cytometry. Antibodies purchased from Biolegend: CD3ε (145-2C11), CD4 (GK1.5), CD11b (M1/70), CD25 (3C7), CD44 (IM7), CD69 (H1.2F3), IFN-γ (XMG1.2), IL-17a (TC11-18H10.1) and T-bet (4B10). Antibodies purchased from BD: GM-CSF (MP1-22E9) and RORγt (Q31-378). Anti-FoxP3 (FJK-16s) was purchased from eBioscience.

### PCR and primers

Quantitation of RNA expression was performed by realtime PCR. Cells were stimulated as described in triplicate and RNA was isolated using the RNeasy Mini kit (Qiagen) following manufacturer’s instructions. Total RNA concentrations were measured using NanoDrop ND-1000 spectrophometer. Reverse transcription reactions in these samples were performed using 1 μg of total RNA with an iScript cDNA Synthesis kit (Bio-Rad). Real-time qPCR was performed with the Roche LightCycler 480 RT PCR Instrument using SYBR Green Mastermix (Applied Biosystems) and the default two-step QRT-PCR program. Amplification curves were evaluated by the comparative Ct analyses. Primers sequences are listed below. The data were collected and analyzed using the comparative cycle threshold method using ribosomal protein S27a as the internal control. Primer sequences: IL12RB1: Forward- TATCCCAGTACCTGTACAAC, Reverse-TCTTCAGACACATTCCAGTC; IL12RB2: Forward- CGGGAAGAGCTCTGGAGAACC Reverse-GCTGACCCAAGAGGAATCACA; IL23R: Forward- ATGGTGTCACGGAGGAATCAC, Reverse- GCATGAGGTTCCGAAAAGCC; and Ribosomal Protein S27a: Forward- GCGAACGAGCAAATCTGGCA, Reverse-GCGGCTCCACCCACGA.

### Cell culture

Cells were isolated from the spleen and lymph nodes of donor mice and single cell suspensions were made by passing the cells through a 70μm mesh, erythrocytes were lysed and cells were washed. Cells were cultured at 2.5x10^5^ cells/ml in in RPMI 1640 that was supplemented with 10% fetal calf serum, L glutamine, 50uM β-mercaptoethanol, 5% NEAA and 5% penicillin/ streptomycin, gentamycin sulfate and 5% CO2 at 37C. Where indicated, cells were stimulated with MOG35-55 peptide, MBP Ac1-11 peptide or monoclonal antibodies against CD3 and CD28. Suboptimal stimulation conditions were anti-CD3 at 1.0 μg/ml and anti-CD28 at 0.5 μg/ml. Optimal stimulation conditions were anti-CD3 at 2.0 μg/ml and anti-CD28 at 1.0 μg/ml. Neutral activation conditions included anti-IL4 (10μg/ml) only. Th1 polarizing cultures included anti-IL4 (10μg/ml) and either recombinant human IL-2 (10 u/ml) or recombinant murine IL-12 (10ng/ml) for 3 days. Th17 polarizing conditions included anti-IL-4 (10μg/ml), Anti-IFNg (10μg/ml) with the indicated combinations of rIL-6 (10ng/mL), rIL-1β (10ng/ml), rIL-23 (10ng/ml), TGFβ1 (2ng/ml) and/or TGFb3 (2ng/ml). The cells were incubated in media containing GSI for at least 30 minutes prior to stimulation. Additional GSI was added to each well daily.

### Flow cytometry

Single cell suspensions were prepared from lymph node, spleen and CNS tissues by mechanical disruption through 70μM mesh. CNS samples were further centrifuged through a 70:30 discontinuous percoll gradient. Nonspecific binding was blocked with Fc receptor blocking agents and stained with fluorophore conjugated mAbs as previously described [42]. Cell viability was detected by labeling dead cells post-culture with Zombie Aqua™ Fixable Viability Kit (Biolegend). Flow cytometry was performed using BD LSR II and Fortessa flow cytometers. Analysis of T cells was performed on FlowJo software v.10 (Treestar). A sequential gating strategy was used to identify single cells, lymphocyte size, live cells and CD4+ T cells. Additional sub-gates were used as indicated in figure legends. Intracellular cytokine and transcription factor detection, cells were activated for four hours with PMA and Ionomycin (Sigma) in the presence of Golgi-stop (BD). The cells were fixed and then permeabilized using the FoxP3 transcription factor buffer kit (eBioscience). T cell proliferation was monitored by labeling cells prior to culture using the Cell Trace Violet Cell Proliferation Kit (Thermofisher). The percentages of T cells within each cell division was identified using sub-gates for each division peak. Division index and Proliferation index were calculated as described [41, 43, 44].

### Statistical analyses

Percent change was calculated by the formula (observed-control)/control. Statistical comparisons were performed using GraphPad Prism 6 software. Correlations between continuous and categorical variables were assessed using the Mann-Whitney U test. The means of two normally distributed samples were compared by Student t-test. Comparisons between multiple groups were performed by one way ANOVA with Tukey’s multiple comparison post-test. P-values <0.05 were considered significant.

## Results

### Effects of blocking γ-secretase on EAE

**Fig 1:**
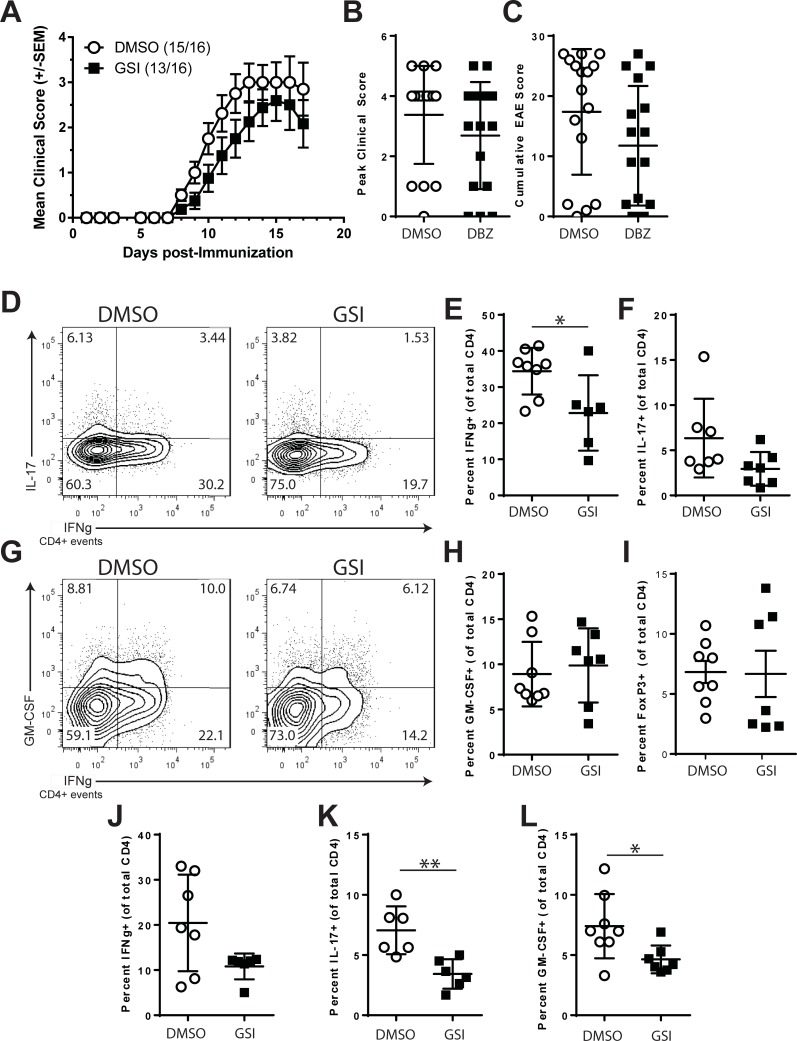
Gamma-secretase inhibitors alter Th1 and Th17 responses but do not inhibit EAE. B6 mice were immunized with MOG35-55 to induce active EAE. Beginning on the day after immunization, the mice were randomized and treated with DMSO or GSI every other day for 18 days. A. Clinical course of EAE. B. Peak clinical score of DMSO and GSI-treated mice. C. Cumulative EAE scores for DMSO and GSI-treated animals. T cell cytokine expression in CNS-infiltrating CD4+ T cells from DMSO or GSI-treated mice following the Peak of EAE. On day 17 post-immunization, CNS cells were isolated and intracellular cytokine staining performed. D. Expression of IL-17 and IFNγ. E. Expression of GM-CSF and IFNγ. Distribution of the percentages of cells expressing IFNγ (F), IL-17 (G), GM-CSF (H) or FoxP3 (I) among CNS-infiltrating CD4+ T cells. J-L, Presence of cytokine expression in the spleens of EAE mice on day 17 post-immunization. Distribution of the percentages of cells expressing IFNγ (J), IL-17 (K), GM-CSF (L). Symbols indicate the percentage of cells from individual mice. Also plotted are the mean and SEM for each treatment group. Open circles indicate DMSO treatment, filled squares indicate treatment with GSI. Error bars represent SEM. Asterisks indicate significant differences (* p<0.05, ** p<0.01 and ***p <0.001).

To understand the functions of PSEN1/γ-secretase in regulating autoimmune T cell responses we utilized small molecule inhibitors of γ-secretase (GSI). We tested the effects of administering Dibenzazepine (DBZ), a potent GSI [45] that has been shown to inhibit γ-secretase function in the CNS [46]. EAE was induced in B6 mice by immunization with MOG35-55 and treated with GSI or vehicle (DMSO) only. Mice were examined for the appearance of EAE symptoms. As shown in Fig 1A, compared to controls, GSI-treated mice developed EAE with similar incidence (DMSO 15/16, GSI 14/16) but only with a modest reduction in symptoms. Treatment with GSI modestly decreased the overall severity of EAE as shown by the mean peak clinical score (Fig 1B) and cumulative EAE score over 17 days (Fig 1C), although these did not achieve statistical significance. Therefore, disruption of γ-secretase by GSI administration failed to significantly impact the severity of EAE in the B6 mouse.

To identify whether GSI treatment altered the immune response in EAE, the CNS-infiltrating T cell populations from DMSO and GSI-treated animals were examined. Representative mice from each treatment group were euthanized on day 17 post-immunization, the CNS was dissected and the immune cell infiltrate was analyzed by flow cytometry. DMSO and GSI-treated animals showed similar numbers of CNS-infiltrating CD4+ T cells (data not shown). Intracellular cytokine staining was used to examine effector T cell subsets isolated from the CNS (Fig 1). CD4+ T cells from control animals expressed IFNγ and IL-17 (Fig 1D), or GM-CSF (Fig 1G). GSI-treated animals showed reductions in the percent of T cells expressing IFNγ (-33.6%, p = 0.0426, Fig 1E) and IL-17 (-53.7%, ns, Fig 1F). GSI treatment did not significantly alter the percentage of CD4 T cells expressing GM-CSF (+9.7%, ns, Fig 1H) or FoxP3 (-2.3%, ns, Fig 1I) in the CNS. Effector T cell subsets were also examined in the spleens of mice treated with DMSO or GSI. Compared to controls, mice treated with GSI showed reduced percentages of CD4 T cells that expressed IFNγ (-47.1%, ns, Fig 1J), IL-17 (-53.3%, p = 0.0043, Fig 1K) and GM-CSF (-37.3%, p = 0.0289, Fig 1L). Thus, although GSI therapy did not significantly alter the course of EAE, it did alter the presence of effector Th1 and Th17 cells in the CNS and peripheral immune organs.

### Impact of GSI treatment on the priming response to MOG35-55

**Fig 2:**
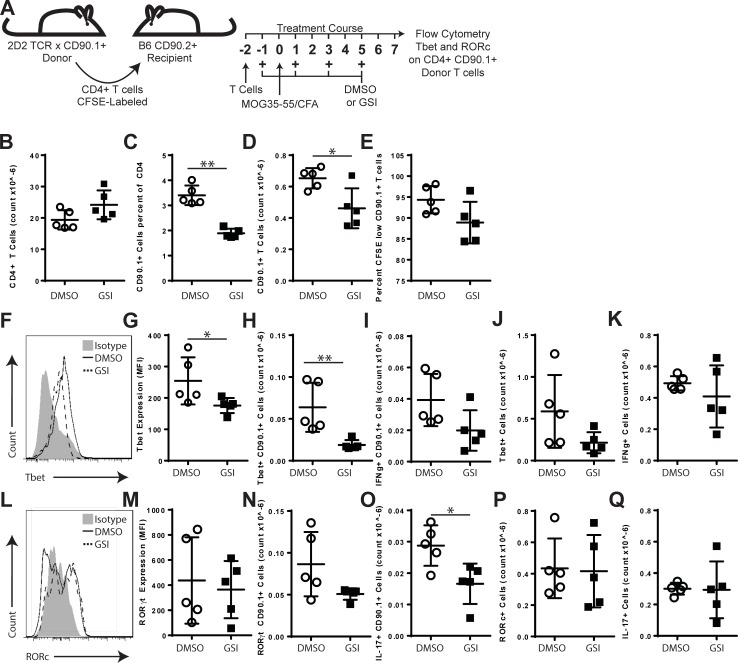
GSI treatment reduces T effector numbers and effector differentiation *in vivo*. A. 2D2 transfer model. T cells from 2D2 TCR transgenic, CD90.1 congenic mice were isolated and transferred on day -1. Mice were immunized with MOG35-55/CFA on day 0. Mice were treated with vehicle (DMSO) or GSI beginning on day -1 as indicated (arrows) and cell responses were detected on day 7 post-immunization. B. Total splenic CD4+ T cell counts. C. Percentages of donor (CD90.1+) among CD4 T cells. D. Total numbers of donor CD4 T cells present in the spleen. E. Percentage of donor T cells that had diluted CFSE. F-K Impact of GSI on Th1 differentiation *in vivo*. F. Histogram overlay of Tbet expression. G. Comparison of Tbet expression (MFI) in donor T cells. H. Numbers of donor cells expressing Tbet. I. the total number of IFNγ+ donor T cells in the spleen. J. Numbers of host-derived CD4+ cells expressing Tbet. K. the total number of IFNγ+ donor T cells in the spleen. L-Q GSI treatment alters Th17 responses L. Histogram overlay of RORγt expression. M. Comparison of RORγt expression (MFI) in donor T cells. N. Numbers of donor cells expressing RORγt. O. the total number of IL-17+ donor T cells in the spleen. P. number of host-derived T cells expressing RORγt. Q. Number of IL-17+ host-derived T cells. Symbols indicate results from individual mice. Open circles indicate treatment with DMSO, filled squares indicate GSI treatment. Error bars indicate standard deviation. Asterisks indicate significant differences (* p<0.05).

Due to the effects of GSI treatment on the CNS-infiltrating T cell populations, we hypothesized that γ-secretase regulates the generation of effector T cell subsets during priming. To directly examine the effects of GSI on MOG35-55-specific T cells we developed a congenic T cell transfer model. In this model, CD4+ T cells were isolated from 2D2 TCR transgenic mice (MOG35-55 specific) that had been bred to express the congenic marker CD90.1. This model allowed for the identification of MOG35-55-specific T cells in flow cytometric analysis using antibodies to CD90.1. The 2D2 CD4+ CD90.1+ T cells were labeled with CFSE and transferred into B6 recipients (Fig 2A). The mice were then treated with GSI or DMSO and immunized with MOG35-55/CFA. On day 7 post-immunization, splenocytes were isolated from each recipient mouse. The numbers and phenotype of the donor T cells was determined by flow cytometry by gating on CD4+, CD90.1+ events while the host T cells were analyzed with CD4+ CD90.1- gated events. Comparisons were made between the DMSO and GSI treatment groups. It was noted that mice treated with GSI had an overall increase in the total number of CD4+ T cells isolated from the spleen relative to controls (+24.6%, ns, Fig 2B). The number of MOG-specific CD90.1+ donor T cells, however, was significantly reduced with GSI treatment as a percentage (-44.5%, p = 0.0079, Fig 2C) and as a total count (-29.3%, p = 0.0317, Fig 2D). In both DMSO- and GSI-treated animals, the majority of donor 2D2 T cells had undergone one or more cell divisions. Donor T cells from GSI-treated animals showed a modest reduction in the percentage of T cells that had diluted CFSE (-5.8%, ns, Fig 2E). These results support a role for γ-secretase in regulating the numbers of MOG35-55-specific T cells generated post-immunization.

We next examined effect of GSI on donor T cell differentiation to the Th1, Th17 and GM-CSF effector phenotypes. Th1 differentiation was assessed by the expression of Tbet and IFNγ. Tbet expression (MFI) was significantly reduced in donor T cells from GSI-treated animals (-31.0%, p = 0.0159, Fig 2F and 2G). GSI-treatment resulted in a reduction in percentage of donor T cells expressing Tbet (-55.5%, p = 0.0159, data not shown) and in the absolute numbers of donor T cells expressing Tbet (-70.5%, p = 0.0079, Fig 2H) in GSI-treated animals. GSI treatment also reduced the percentage of donor T cells expressing IFNγ (-27.5%, ns, data not shown) and the total number of donor T cells expressing IFNγ (-49.4%, ns, Fig 2I). The effects of GSI on Th1 differentiation were also examined on CD4+ T cells from the host. GSI-treated animals were found to have a trend toward reduced numbers of host-derived Th1 cells that expressed Tbet (-63.7%, ns, Fig 2J) and IFNγ (-17.0%, ns, Fig 2K).

The effects of GSI on Th17 cell differentiation were determined by measuring the expression of RORγt and IL-17. Compared to controls, GSI-treatment did reduce Th17 differentiation as assessed by RORγt expression (-16.7%, ns, Fig 2L and 2M), the percentage of donor T cells that were positive for RORγt (-11.5%, ns, data not shown) and the total number of donor T cells expressing RORγt was reduced (-41.2%, ns, Fig 2N). GSI treatment was also found to reduce the percentage of donor T cells expressing IL-17 (-17.1%, ns, data not shown) and significantly impacting the total number of donor T cells expressing IL-17 (-42.3%, p = 0.0317, Fig 2O). The effects of GSI on Th17 differentiation were also examined on CD4+ T cells from the host. GSI-treated animals were found to have comparable numbers of host-derived T cells that expressed RORγt (-4.2%, ns, Fig 2P) and IL-17 (-2.4%, ns, Fig 2Q).

The data demonstrate that GSI treatment qualitatively and quantitatively altered the composition of the MOG-specific T cell responses *in vivo* by reducing the numbers responding T cells and by altering the differentiation of Th1, and Th17 effector T cell subtypes *in vivo*.

### GSI inhibit Th1 differentiation and T cell activation *in vitro*

**Fig 3:**
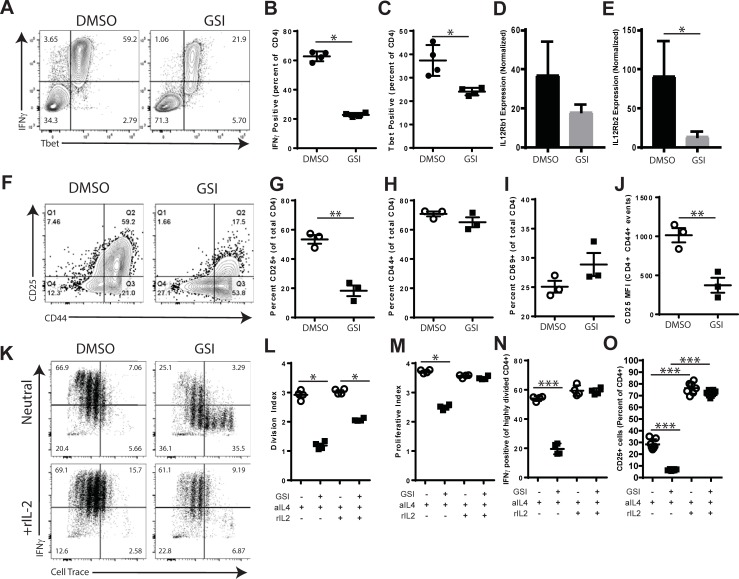
GSI treatment inhibits effector differentiation, activation and proliferation *in vitro*. A-E. Effects of GSI on Th1 differentiation. Splenocytes were stimulated *in vitro* with anti-CD3 and anti-CD28 for 72 hours in the presence of anti-IL-4 and either DMSO or GSI. A-C. IFNγ and Tbet expression were determined by intracellular staining and flow cytometry. A. Representative flow cytometry plots showing IFNγ and Tbet expression. B. IFNγ expression was measured by intracellular flow cytometry. C. Tbet expression was measured by intracellular flow cytometry. D and E. Expression analysis of IL12Rβ1 and IL12Rβ2. Total cellular RNA was isolated from each culture and target gene expression determined by real time PCR. D. Expression of IL12Rβ1. E. Expression of IL12Rβ2. F-J. Effects of GSI on T cell activation. Splenocytes from 2D2 TCR transgenic mice were activated *in vitro* with MOG35-55 peptide in the presence of DMSO or GSI. At 72 hours of stimulation, flow cytometry was used to measure expression of the activation markers CD25, CD44 and CD69. F. Representative flow cytometry plots showing CD25 and CD44 expression. G. The percentage of T cells expressing CD25. H. Expression of CD44 by T cells. I. The percentages of T cells expressing CD69. J. Quantitation of CD25 expression on activated T cells (CD4 and CD44 gated events). K-N Effects of GSI on T cell proliferation. Splenocytes were labeled with Cell-trace and stimulated for 96 hours with antibodies to CD3 and CD28 in the presence of DMSO or DBZ. Th1 differentiation was promoted by the addition of anti-IL-4 alone (neutral), or in combination with IL-2. K. Proliferation and intracellular IFNγ staining were detected by flow cytometry. L and M. Division index and Proliferation index were calculated using gates to measure the percentages of T cells within each cell division. O. Expression of IFNγ by T cells that had undergone 4 or more cell divisions. P. Expression of CD25 by T cells cultured in the absence or presence of IL-2. Flow cytometry plots are gated on live CD4+ T cells. Results shown are representative of at least two experiments. The numbers in FACS plots indicate cell percentages within each quadrant. Individual symbols indicate results from replicate wells. Open circles indicate treatment with DMSO, filled squares indicate GSI treatment. Error bars indicate SEM. Asterisks indicate significant differences (* p<0.05, ** p<0.01 and ***p <0.001).

*In vitro* models were next used to examine the role of γ-secretase in T cell differentiation, activation and proliferation. We first examined Th1 differentiation in neutral conditions. T cells were activated in bulk splenocytes cultures in the presence of anti-IL-4 by stimulation with optimal concentrations of antibodies to CD3 and CD28. DMSO or GSI were added to the each well. Intracellular flow cytometry was used to detect IFNγ and Tbet expression at 72 hours post-stimulation (Fig 3A). T cells activated in the presence of GSI showed reduced expression of IFNγ (Fig 3B, -64.5%, p = 0.0286) and Tbet (Fig 3C, -33.8%, p = 0.0286). In parallel experiments, T cells activated in the presence of GSI also showed a reduction in the expression level of IL12Rβ1 (-50.7%, ns, Fig 3D) and IL12Rβ2 (-84.6%, p = 0.0416, Fig 3E) as measured by quantitative PCR.

The fact that GSI treatment altered the expression of multiple Th1-associated genes suggested that γ-secretase may regulate T cell activation. To examine the effects of GSI on the T cell activation process, splenocytes from 2D2 TCR transgenic mice were stimulated with MOG35-55. As shown in Fig 3, activation was measured by surface expression of CD25, CD44 and CD69. At early time points, cells cultured in GSI showed reduced surface expression of CD25 (-43.6%, ns, data not shown) and CD44 (-12.4%, p = 0.0127, data not shown). By contrast, DMSO and GSI-treated cells expressed CD69 at similar percentages and MFI (data not shown). In 72 hour cultures (Fig 3F), the percentage of T cells expressing CD25 was found to be significantly reduced (-65.8%, p = 0.0017, Fig 3G). CD44 was slightly reduced at 72 hours (-8.1%, ns, Fig 3H). At 72 hours, CD69 expression was increased in GSI-treated cells (+15.2%, ns, Fig 3I) compared to DMSO controls. Interestingly, CD25 expression (MFI) was significantly reduced (-63.2%, p = 0.0084, Fig 3J) on gated CD44+ T cells suggesting that GSI selectively impairs CD25 expression. Thus we conclude that γ-secretase-dependent signals regulate T cell responses by selectively regulating the expression of Th1-promoting cytokine receptors including CD25, IL12Rβ1 and IL12Rβ2 by activated CD4+ T cells.

Next, we examined the effects of GSI on T cell proliferation and Th1 differentiation using an *in vitro* proliferation assay. Splenocytes were labeled with cell-trace violet and T cells were stimulated with anti-CD3 with either DMSO or GSI (Fig 3K). Proliferation was measured by dye dilution using gates for each cell division and compared between groups by calculating the division index (DI) and proliferative index (PI). CD4+ cells from GSI-treated wells showed significant reductions in both the DI (-59.3%, p = 0.0286, Fig 3L) and PI (-32.9%, p = 0.0286, Fig 3M). This is consistent with a previously described role for Notch in regulating T cell proliferation [47]. Given the defects in CD25 expression, we also tested whether IL-2 would alter the effects of GSI on T cell proliferation. When cultured in the presence of IL-2, GSI treatment significantly reduced proliferation as measured by DI (-32.0%, p = 0.0286, Fig 3L) but no significant differences were detected in PI (-1.9%, ns, Fig 3M). These results are consistent with γ-secretase regulating the initial cell divisions and the maintained progression through cell cycle. IL-2 enhanced the progression of cell cycle (PI) but did not alter the initial divisions calculated into the DI.

Given that GSI-induced defects in proliferation might reduce the overall percentage of cytokine producing T cells, the relationship between T cell proliferation and IFNγ expression was explored. As shown in Fig 3, quadrant gates were used to identify T cells that had undergone four or more divisions (Fig 3K left quadrants) and T cells that expressed IFNγ (Fig 3K top quadrants). The percentage of IFNγ-expressing T cells among the highly proliferated T cell fraction was then calculated. As shown in Fig 3N, the expression of IFNγ in highly proliferated T cells was significantly reduced by GSI as compared to DMSO-treated controls (-63.8%, p<0.0001). The addition of IL-2 augmented proliferation and restored IFNγ expression in GSI-treated cultures to the level of controls (-0.9%, ns). These results demonstrate that inhibitory effects of GSI on IFNγ-expression were not due to impaired proliferation. The data support a role for γ-secretase-dependent signals in regulating the outgrowth of Th1 effectors and Th1 differentiation by modulating IL-2 signaling.

The fact that GSI-treated cells responded to IL-2 was surprising considering that CD25 is the high-affinity component of the IL-2 receptor. To explore the relationship between γ-secretase and IL-2 responsiveness, we re-examined the effects of GSI on CD25 expression. In neutral conditions, GSI significantly inhibited CD25 expression (-77.3%, p<0.0001, Fig 3O). The addition of recombinant IL-2 significantly increased the expression of CD25 in DMSO (+168.9%, p<0.0001) and GSI-treated cells (+1011.9%, p<0.0001). Compared to DMSO +IL-2-treated controls, cells that were activated with GSI + IL-2 showed a small but significant reduction in CD25 expression (-5.9%, p = 0.0428). Therefore, GSI inhibited CD25 expression when elicited by TCR signals but did not suppress CD25 expression induced by IL-2R signals.

### γ-secretase regulates Th1 differentiation in neutral but not strong polarizing conditions

**Fig 4:**
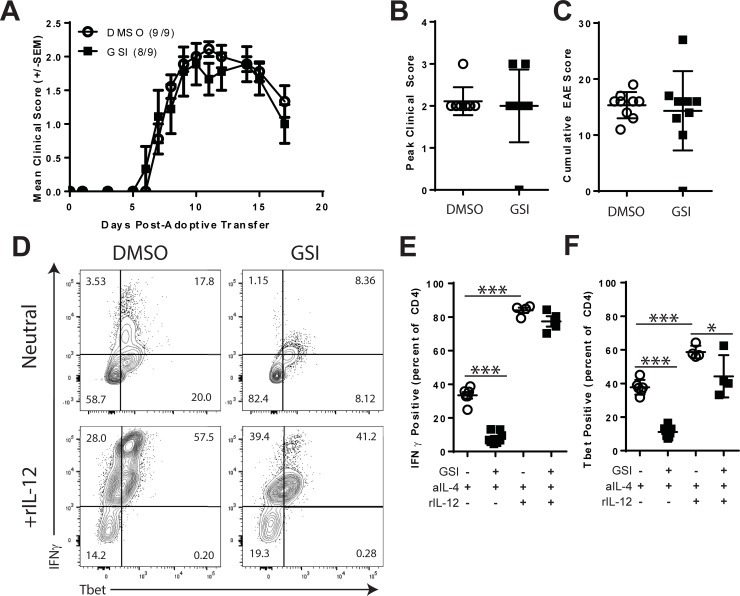
Impact of GSI on Th1 effector differentiation. A-C. Effects of GSI on the generation of encephalitogenic Th1 T cells. T cells from MBP1-11 TCR transgenic mice were activated *in vitro* with MBP ac1-11 peptide and IL-12. Cell cultures were stimulated in the presence of DMSO or GSI. 8x10^6^ T cells were transferred into B10.Pl recipients and mice were graded for the development of EAE. A. Mean EAE disease course. B. Peak clinical score. C. Cumulative EAE score. D-F. Effects of GSI on Th1 polarization with IL-12. Splenocytes were stimulated *in vitro* with anti-CD3 and anti-CD28 for 72 hours in the presence of anti-IL-4 alone with IL-12. IFNγ and Tbet expression was measured by intracellular flow cytometry at 72 hours using gates for live CD4+ T cell events. E. Percentages of IFNγ expressing CD4+ T cells. F. Percentages of T cells expressing Tbet. Results shown are representative of at least two experiments. Open circles indicate DMSO treatment. Filled squares indicate GSI. Symbols represent individual mice (panels B and C) or replicate cultures (panels E and F). The numbers in FACS plots indicate cell percentages within each quadrant. Asterisks indicate statistically significant differences between groups (* p<0.05, ** p<0.01 and ***p <0.001).

We next sought to identify whether GSI treatment altered the pathogenicity of Th1 T cells using an adoptive transfer model of EAE. T cells from MBP1-11 TCR transgenic mice were activated *in vitro* with MBP Ac1-11 peptide and polarized to a Th1 phenotype with IL-12 in the presence of either DMSO or GSI. On day 4 of culture, T cells were transferred into B10.PL recipients. Unexpectedly, mice that received Th1 T cells from either DMSO or GSI-treated cultures developed EAE (Fig 4A) with similar incidence and severity (Fig 4B and 4C). *In vitro* activation in the presence of GSI did not alter the generation of pathogenic effector Th1 T cells *in vitro*. This result was unanticipated because of the inhibitory effects of GSI on Th1 differentiation in non-polarizing conditions (Fig 3), however, this adoptive transfer model was dependent on Th1 polarization with IL-12. Therefore, we hypothesized that GSI regulated Th1 differentiation in weak but not strong Th1 conditions.

The effects of GSI on IL-12 driven Th1 polarization were tested. T cells were activated *in vitro* in the absence or presence of GSI and anti-IL-4 alone (neutral) or with recombinant IL-12 (Fig 4D). For these experiments, sub-optimal doses of CD3 and CD28 were used to assess the impact of GSI on IL-12-stimulated Th1 polarization. Under neutral conditions, Th1 differentiation was significantly inhibited by GSI with reductions in the expression of IFNγ (-75.7%, p<0.0001, Fig 4E) and Tbet (-70.4%, p<0.0001, Fig 4F). Consistent with its role in Th1 differentiation, IL-12 significantly increased IFNγ (+151.3%, p<0.0001) and Tbet (+55.6%, p = 0.0003) expression in DMSO-containing wells and in GSI-containing cultures (IFNγ, +854.5%, p<0.0001, Fig 4E and Tbet, +296.2%, p<0.0001, Fig 4F). Compared to controls that had been treated with IL-12 and DMSO, cells cultured in the presence of GSI and IL-12 expressed lower levels of IFNγ (-7.8%, ns, Fig 4E) and Tbet (-24.6%, p = 0.0185, Fig 4F). Therefore, GSI treatment failed to inhibit the generation of Th1 effector responses when T cells were activated in the presence of IL-12.

### γ-secretase regulates the differentiation of pathogenic Th17 cells *in vitro*

**Fig 5:**
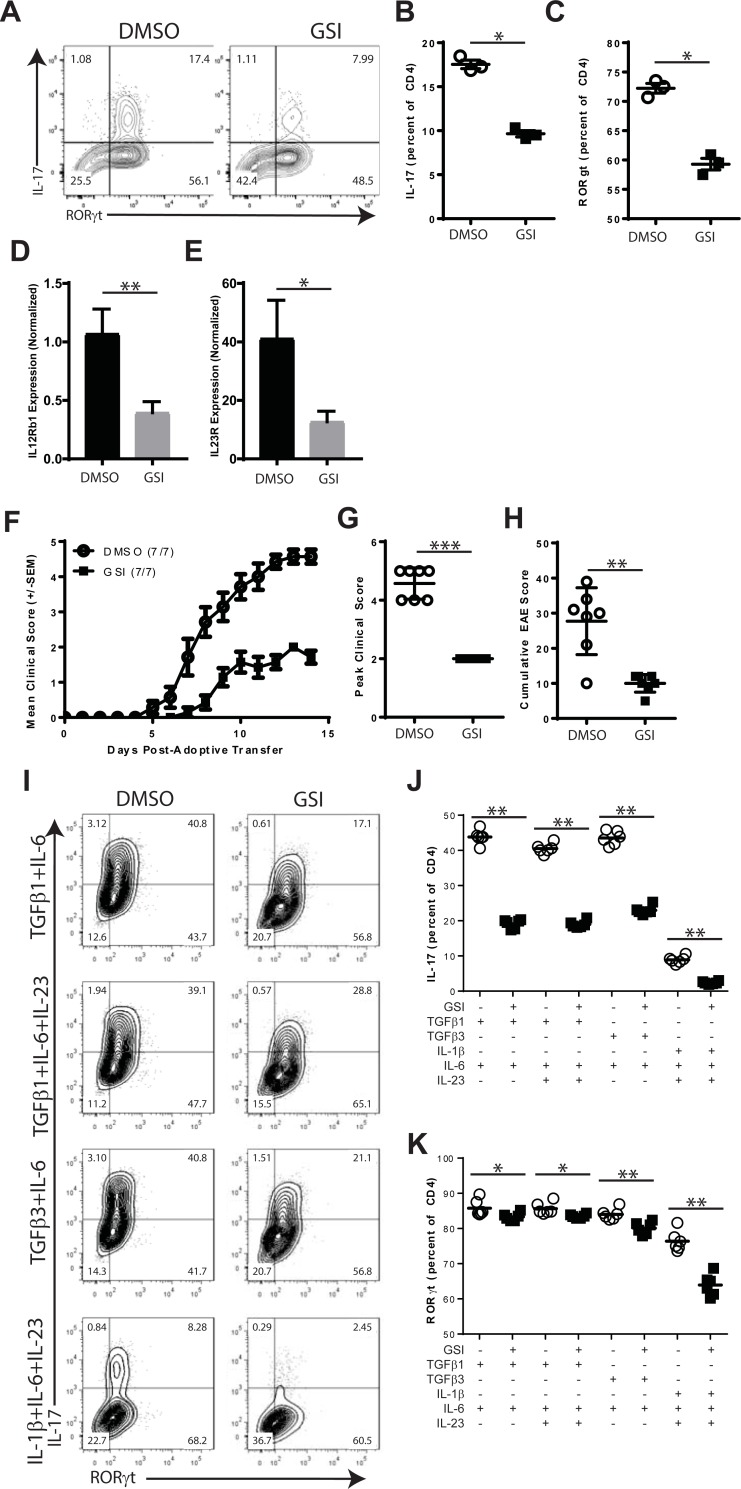
The effects of GSI on Th17 differentiation. A-E. Effects of GSI on Th17 differentiation. Splenocytes were stimulated *in vitro* with anti-CD3 and anti-CD28 with TGFβ1 and IL-6 for 72 hours in the presence of either DMSO or GSI. Intracellular flow cytometry was used to measure IL-17 and RORγt expression. A. Representative flow cytometry plots showing IL-17 and RORγt expression. B. IL-17 expression was measured by intracellular flow cytometry. C. RORγt expression was measured by intracellular flow cytometry. D and E. Total cellular RNA was isolated from each culture and realtime PCR was performed to identify the expression of IL12Rβ1 and IL23R. D. Expression of IL12Rβ1. E. Expression of IL23R. F-H. Effects of GSI on encephalitogenic Th17 T cell responses. Splenocyte cultures from MBP1-11 TCR transgenic mice were activated *in vitro* with MBP ac1-11 peptide and polarized with TGFβ1, IL-6 and IL-23. Cells were cultured for 72 hours in the presence of DMSO or GSI. 8x10^6^ T cells were transferred into B10.Pl recipients and mice were graded for the development of EAE. F. Mean EAE disease course. G. Peak clinical score. H. Cumulative EAE score. I-K. Effects of GSI on Th17 polarization with Splenocytes were stimulated *in vitro* with anti-CD3 and anti-CD28 for 96 hours in four Th17-inducing conditions: TGFβ1 and IL-6; TGFβ1, IL-6 and IL-23; TGFβ3 and IL-6; or IL-1β, IL-6 and IL-23. IL-17 and RORγt expression was measured by intracellular flow cytometry at 96 hours. I. Representative FACS plots for each polarizing condition. J. Percentages of IL-17 expressing CD4+ T cells for each polarizing condition. K. Percentages of T cells expressing RORγt for each polarizing condition. Results shown are representative of at least two experiments. Open circles indicate DMSO treatment. Filled squares indicate GSI. Symbols represent individual mice (panels B and C) or replicate cultures (panels E and F). Flow plots are gated on live CD4+ events. The numbers in FACS plots indicate cell percentages within each quadrant. Asterisks indicate statistically significant differences between groups (* p<0.05, ** p<0.01 and ***p <0.001).

Th17 differentiation was next addressed using *in vitro* stimulation cultures. Splenocytes were activated with anti-CD3 and anti-CD28 in the presence of anti-IL-4, TGFβ1 and IL-6. DMSO or GSI were added to each culture. On day 4, flow cytometry was used to assess the expression of IL-17 and RORγt (Fig 5A). T cells cultured in the presence of GSI showed reduced the percentages of cells expressing IL-17 (-81.8%, p = 0.011, Fig 5B) and RORγt (-72.2%, p = 0.019, Fig 5C). GSI treatment also reduced the expression of IL12Rβ1 (-63.0%, p = 0.0073, Fig 5D) and IL23R (-69.1%, p = 0.022, Fig 5E). The observed reductions in IL23R expression are consistent with defects in the Notch pathway [48].

The effects of GSI on the development of pathogenic Th17 responses pathogenicity were examined using the MBP1-11-T cell transfer model. Naïve T cells from MBP1-11-specific TCR transgenic donors were isolated and stimulated with MBP ac1-11in the presence of the polarizing cytokines IL-6, TGFB1 and IL-23 [49] in the presence of DMSO or GSI. Equal numbers of activated T cells were transferred into syngeneic B10.PL recipients. Th17 T cells from DMSO-treated cultures elicited severe EAE (Fig 5F). By contrast, mice that received GSI-treated T cells developed EAE with reduced severity including significant reductions in mean peak clinical score (-56.3%, p = 0.0006, Fig 5G), reduced cumulative severity (63.9%, p = 0.0070, Fig 5H) and delayed day of onset (p = 0.0146). We conclude that γ-secretase is critical to the development of encephalitogenic Th17 effector T cells.

We next sought to determine whether GSI treatment regulated Th17 differentiation induced by different cytokine combinations. As shown in Fig 5, T cells were stimulated with four different cytokine mixtures to induce Th17 differentiation: TGFβ1 and IL-6; TGFβ1 with IL-6 and IL-23; TGFβ3 and IL-6; or IL-1β with IL-6 and IL-23. Th17 polarization was measured by flow cytometry (Fig 5I) to detect IL-17 (Fig 5J) and RORγt (Fig 5K). In cultures containing TGFβ1 and IL-6, the addition of GSI was found to significantly reduce the expression of IL-17 (-56.3%, p = 0.0022) and RORγt (-2.86%, p = 0.0238). When polarization was induced with TGFβ1, IL-6 and IL-23, the presence of GSI reduced the percentage of T cells expressing IL-17 (-53.1%, p = 0.0022) and RORγt expression (-2.49%, p = 0.0043). GSI treatment reduced the expression of IL-17 (-47.2%, p = 0.0022) and RORγt (-4.64%, p = 0.0022) when T cells were polarized with TGFβ3 and IL-6. GSI treatment also significantly inhibited IL-17 (-73.2%, p = 0.0022) and RORγt (-16.3%, p = 0.0022) when stimulated by IL-1β, IL-6 and IL-23. Therefore, GSI treatment altered the expression of IL-17 and had a small impact on RORγt expression during T cell polarization with different cytokines.

### T cell-specific deletion of PSEN1 does not alter EAE

**Fig 6:**
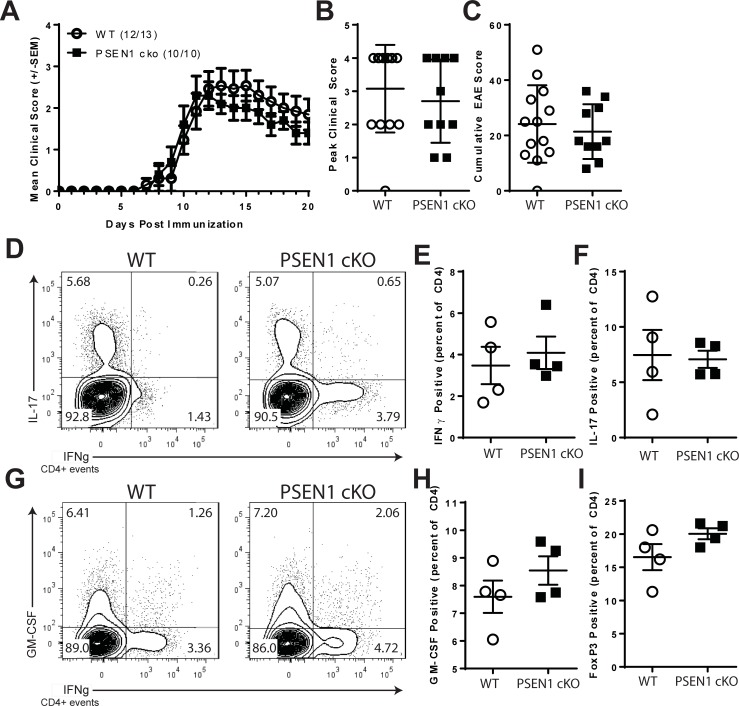
PSEN1 cKO mice are susceptible to EAE. WT and PSEN1 cKO mice were immunized with MOG35-55 to induce active EAE. A. Clinical course of EAE. B. Peak clinical score of EAE mice. C. Cumulative EAE scores for DMSO and GSI-treated animals. T cell cytokine expression in CNS-infiltrating CD4+ T cells from DMSO or GSI-treated mice following the Peak of EAE. On day 17 post-immunization, CNS cells were isolated and intracellular cytokine staining performed. D. Expression of IL-17 and IFNγ. E. Expression of GM-CSF and IFNγ. Distribution of the percentages of cells expressing IFNγ (F), IL-17 (G), GM-CSF (H) or FoxP3 (I) among CNS-infiltrating CD4+ T cells. Symbols indicate the percentage of cells from individual mice. Open circles indicate results from WT and filled squares indicate PSEN1 cKO mice. Error bars represent SEM. Asterisks indicate significant differences (* p<0.05, ** p<0.01 and ***p <0.001).

T cell-specific role of PSEN1/γ-secretase in regulating EAE was next examined. Mice expressing the conditional allele of PSEN1 (PSEN1 lox/lox) [50] were obtained and bred to the CD4-Cre transgenic background. The mice were intercrossed to create CD4-Cre+ PSEN1 lox/lox (PSEN1 cKO) and PSEN1 lox/lox (WT) controls. WT and PSEN1 cKO mice were immunized with MOG35-55 to induce EAE. As shown in Fig 6, EAE was comparable in WT and PSEN1 cKO mice. PSEN1 cKO mice had a consistently reduced severity of EAE, however, no significant differences in disease course (Fig 6A), peak clinical score (Fig 6B), cumulative EAE score (Fig 6C) or day of onset (data not shown) were detected.

The role of PSEN1 in T cell effector differentiation was examined *in vivo*. WT and PSEN1 cKO mice were immunized with MOG35-55 and on day 7 intracellular cytokine staining was performed on T cells in the lymph nodes draining the sites of immunization (Fig 6D and 6G). Although, changes were detected in the percentages of T cells expressing IFNγ (+17.63%, ns, Fig 6E), IL-17 (-5.16%, ns, Fig 6F) or GM-CSF (+12.51%, ns, Fig 6G and 6H), however, these were not significantly changed compared to WT controls. An increase in the percentage of T cells expressing FoxP3 was also found in lymph node cells from PSEN1 cKO mice (+21.3%, ns, Fig 6I).

### Impaired Th1 and Th17 differentiation in the absence of PSEN1

**Fig 7:**
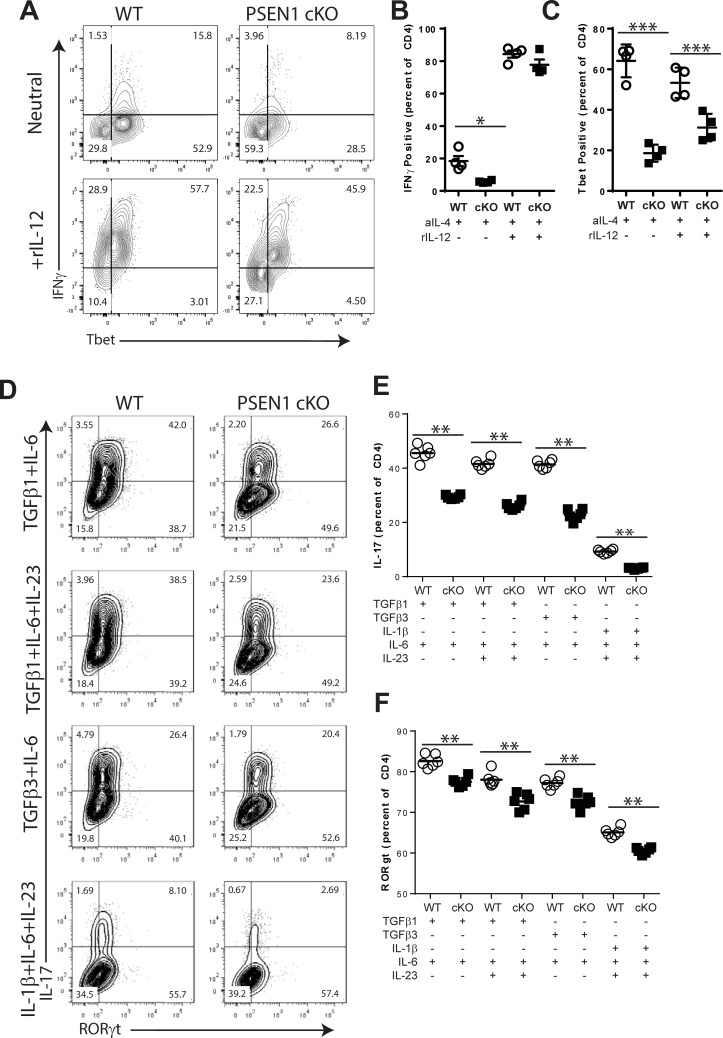
Altered Th1 and Th17 differentiation in the absence of PSEN1 expression. A-C. Impact of PSEN1 on Th1 differentiation *in vitro*. Splenocytes isolated from WT and PSEN1 cKO donors were stimulated *in vitro* with anti-CD3 and anti-CD28 for 72 hours in the presence of anti-IL-4 alone or together with recombinant IL-12. IFNγ and Tbet expression were measured by intracellular flow cytometry at 72 hours. A. Representative FACS plots. B. Percentages of IFNγ expressing CD4+ T cells was determined in replicate cultures containing DMSO, GSI and either neutral, IL-2 or IL-12 polarizing conditions. C. Percentages of T cells expressing Tbet was determined under each culture condition. D-F. PSEN1 regulates Th17 polarization *in vitro*. Splenocytes isolated from WT and PSEN1 cKO donors were stimulated *in vitro* with anti-CD3 and anti-CD28 for 96 hours in four Th17-inducing conditions: TGFβ1 and IL-6; TGFβ1, IL-6 and IL-23; TGFβ3 and IL-6; or IL-1β, IL-6 and IL-23. IL-17 and RORγt expression was measured by intracellular flow cytometry at 96 hours. D. Representative FACS plots for each polarizing condition. E. Percentages of IL-17 expressing CD4+ T cells for each polarizing condition. F. Percentages of T cells expressing RORγt for each polarizing condition. Results shown are representative of at least two experiments. Open circles identify cells from WT donors, filled squares are from PSEN1 cKO donors. Symbols represent replicate cultures. Flow plots are gated on live CD4+ events. The numbers in FACS plots indicate cell percentages within each quadrant. Asterisks indicate statistically significant differences between groups (* p<0.05, ** p<0.01 and ***p <0.001).

WT and PSEN1 cKO T cells were activated with sub-optimal concentrations of antibodies against CD3 and CD28 in neutral conditions or with added IL-12. Th1 differentiation was measured by intracellular flow cytometry to detect IFNγ and Tbet expression after 72 hours of culture (Fig 7A). As shown in Fig 7B, T cells from PSEN1 cKO donors had a significant reduction in IFNγ Tbet expression following culture in neutral conditions (-69.0% p = 0.0183) but when cultured with recombinant IL-12, IFNγ expression was restored to the level of controls (-7.8%, ns). By contrast, Tbet expression was significantly reduced in PSEN1 cKO T cells cultured in neutral (-71.0%, p<0.0001, Fig 7C) and in IL-12 culture conditions (-41.4%, p = 0.0008). Thus, PSEN1-dependent signals regulate Th1 differentiation in a T cell-intrinsic manner.

The role of PSEN1 on Th17 differentiation was also examined. Cells from WT and PSEN1 cKO donors were stimulated *in vitro* with anti-CD3 and anti-CD28 mAb and Th17 differentiation was elicited by the addition of polarizing cytokines (Fig 7D). As shown in Fig 7E, the percentage of PSEN1 cKO T cells expressing IL-17 was significantly reduced in cultures containing TGFβ1 and IL-6 (-35.5%, p = 0.0022); TGFβ1, IL-6 and IL-23 (-37.3%, 0.0022); TGFβ3 and IL-6 (-45.1%, p = 0.0022); or IL-1β, IL-6 and IL-23 (-66.6%, 0.0022). As shown in Fig 7F, T cells from PSEN1 cKO mice showed modest reductions in the expression of RORγt following stimulation with TGFβ1 and IL-6 (-6.19%, p = 0.0022); TGFβ1, IL-6 and IL-23 (-6.85%, p = 0.0022); TGFβ3 and IL-6 (-6.04%, p = 0.0022); or IL-1β, IL-6 and IL-23 (-6.91%, p = 0.0022). Thus, PSEN1 regulates Th17 differentiation in a T cell-intrinsic manner.

### T cell-intrinsic PSEN1 regulates proliferation but not activation

**Fig 8:**
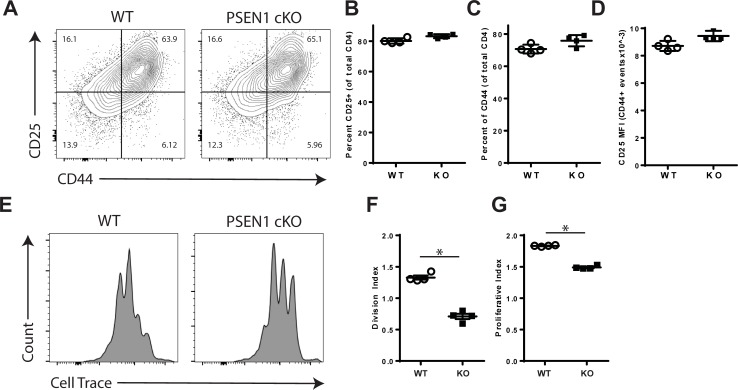
PSEN1 regulates T cell proliferation but not activation. Panels A-D. Activation in PSEN1 cKO T cells. Splenocytes from WT and PSEN1 cKO mice were isolated and stimulated with soluble anti-CD3 and anti-CD28 antibodies. A. At 72 hours of culture T flow cytometry was performed to measure the expression of CD44 and CD25 on CD4+ T cells. B. Percentage of CD4+ T cells that express CD25. C. Quantitation of CD44 expression on CD4+ gated T cells D. CD25 surface expression level (MFI) of activated T cells that express both CD4 and CD44. Panels E-G. T cell proliferative responses. E. Cell Trace-labeled splenocytes were activated with anti-CD3 and anti-CD28 for 72 hours. Flow cytometry was performed to measure dye dilution by CD4+ gated events. F. The division index was calculated for WT and PSEN1 cKO T cells. G. The proliferation index was calculated for WT and PSEN1 cKO T cells. Mean expression data are plotted+/- SEM. Open circles and filled squares indicate WT and PSEN1 cKO T cells, respectively. Asterisks indicate significant differences (* p<0.05, ** p<0.01 and ***p <0.001).

*In vitro* stimulation cultures were next used to examine the impact of PSEN1 on T cell activation, Th1 differentiation and proliferation. For these experiments, splenocytes were isolated from WT and PSEN1 cKO mice and T cells were stimulated with antibodies to CD3 and CD28. First, the T cell activation process was examined by measuring the cell-surface expression CD25 and CD44 by flow cytometry. Following stimulation T cells from WT and PSEN1 cKO mice expressed similar levels of surface CD25 and CD44 at 24 hours (data not shown) and at 72 hours (Fig 8A–8D).

Due to the inhibitory effects of GSI on T cell proliferation, experiments were also performed using cells from PSEN1 cKO mice. WT and PSEN1 cKO splenocytes were isolated, labeled with Cell Trace and stimulated *in vitro* with CD3 and CD28 antibodies. T cell proliferation was examined at 72 hours post-stimulation by flow cytometry. As shown in Fig 8E, T cells isolated from PSEN1 cKO mice showed an increased number of cells that had not divided and the average extent of division was reduced as compared to T cells isolated from WT controls. When analyzed, PSEN1 cKO T cells showed significant reductions in both DI (-46.6%, p = 0.0286, Fig 8F) and PI (-18.7%, p = 0.0286, Fig 8G).

## Discussion

Notch and γ-secretase have been proposed as potential therapeutic targets for inflammatory disorders including MS. This project was designed to test the effects of disrupting PSEN1-dependent γ-secretase in regulating autoimmune T cell responses in MOG35-55 induced EAE. The effects of systemic GSI treatment and T cell-specific PSEN1 deficiency were tested *in vivo* and *in vitro*. *In vivo* GSI treatments and PSEN1 deletion had modest effects on autoimmunity in the EAE model. In addition, γ-secretase and PSEN1 were found to be dispensable for encephalitogenic effector T cell responses in EAE. We identified a role for γ-secretase and PSEN1 in regulating T cell activation, proliferation and T effector differentiation *in vitro*, however, the effects of disrupting PSEN1/γ-secretase *in vivo* are much less pronounced. Thus we conclude that while targeting γ-secretase has beneficial effects on some aspects of T cell responses, it is not an effective method to interrupt autoimmunity.

### Effects of γ-secretase on EAE

In B6 mice, immunization with MOG35-55 elicits diverse T cell responses involving Th1, Th17 and GM-CSF-producing cells, each of which can be encephalitogenic (reviewed in [51]). Unexpectedly, GSI administration post-immunization produced only a modest reduction in EAE severity (Fig 1). The observed effects of GSI administration were consistent with previous reports of GSI therapy in PLP139-151-induced models of EAE in SJL mice [30, 34, 52]. Interestingly, mice lacking PSEN1 expression in T cells also showed very little difference in the course of EAE (Fig 6). This suggests that PSEN1 expression by T cells is dispensable for the development of autoimmunity. Due to the role of γ-secretase in the Notch signaling pathway, it is surprising that the phenotype of the PSEN1 cKO mice does not closely match that of mice that lack other critical Notch pathway components. Mice that lack the expression of RBP-jk [48] or mice that express the dominant negative form of Mastermind 1 (DNMAML1) [53], both of which developed EAE with reduced severity.

Despite the modest effects on EAE, GSI treatment was associated with significant changes in the composition of the CNS-infiltrating and a trend toward reduced effector T cell populations in the spleen (Fig 1). In the CNS, GSI treated animals showed reductions in the percentages of CD4 T cells expressing IFNγ and IL-17 in the CNS.

### γ-secretase promotes T cell activation proliferation

T cell responses are initiated through the process of activation and proliferation. *In vivo* GSI treatment was found to reduce the numbers of MOG-specific T cells generated following immunization (Fig 2). This finding suggested a role for γ-secretase in regulating the processes of T cell activation and proliferation. Following stimulation in the presence of GSI, T cells expressed lower levels of CD25 but not CD44 or CD69 suggesting that γ-secretase does not directly inhibit all TCR signals but selectively regulates CD25 expression (Fig 3). The functions of γ-secretase in regulating CD25 expression are complex. First, GSI treatment inhibited CD25 expression downstream of TCR/CD28 signals but not in the presence of recombinant IL-2. Second, T cells from PSEN1 cKO did not show impaired expression of CD25 (Fig 8), suggesting that γ-secretase influences CD25 through the TCR/CD28 pathways but not in the presence of IL2R/STAT5 signals. The differences in CD25 expression between GSI treated and PSEN1 cKO T cells identify that the inhibitory effects of γ-secretase on CD25 expression are not T cell intrinsic. Additionally, *in vitro* assays showed that GSI reduced the extent of proliferation by altering proliferation as measured by DI and PI (Fig 3). Similarly, T cells from PSEN1cKO donors also showed inhibited DI and PI suggesting that PSEN1 expression by T cells influences T cell proliferation (Fig 8).The fact that both metrics were altered by GSI treatment and PSEN1 deletion indicates a role for γ-secretase dependent signals in initial cell division and subsequent mitotic events. Addition of IL-2 to the in vitro activation cultures restored the PI to the level of controls suggesting that the effects of γ-secretase on cell cycle progression may be linked to defects in IL-2 signaling. Our data support a model where PSEN1 and γ-secretase-dependent signals promote but are not required for T cell activation and proliferation, these effects were overcome by the presence of IL-2.

### Th1 differentiation is regulated by γ-secretase

The relationship between γ-secretase and Th1 differentiation is multifaceted. *In vivo* treatment with GSI reduced the percentage of T cells expressing IFNγ in the CNS and periphery (Fig 1). MOG-specific T cells expressing Tbet or IFNγ were reduced as a percentage (data not shown) and as a total number (Fig 2) following immunization. These results are similar to treatment with GSI in other models [30]. By contrast, PSEN1 cKO mice did not show altered Th1 responses (Fig 6). Thus, *in vivo* treatment with GSI and T cell-specific conditional knockout animals have different effects with relationship to Th1 differentiation. The differences between GSI treatment and T cell-specific PSEN1 conditional knockout mice may be model-specific in that GSI treatment has the potential to impact numerous cell types including T cells while CD4-Cre driven deletion of PSEN1 only alters γ-secretase in T cells. In this scenario, *in vivo* GSI administration would regulate γ-secretase and Notch signals in other critical cell types. Experimental evidence demonstrates a role for Notch signals in regulating the pro-Th1 functions of myeloid cells. Notch signals were found to be directly involved in the expression of IL-12 by macrophages [54] and monocyte-derived dendritic cells [55]. In addition, myeloid cell-specific Notch1 signals have been found to influence the severity of EAE by controlling the generation of Th1 and Th17 T cells in vivo [56].

There are ample data to support a role for Notch signals in Th1 differentiation *in vitro* [22, 30, 57–62]. Th1 differentiation *in vitro* was inhibited by treatment with GSI (Fig 3) under neutral conditions but was restored by exogenous IL-2 (Fig 3) or IL-12 (Fig 4). T cells polarized with IL-12 in the presence of GSI were able to induce EAE (Fig 4). IFNγ and Tbet expression was reduced in T cells from PSEN1 cKO donors (Fig 7) when cultured with neutral conditions, however, the addition of IL-2 or IL-12 restored expression of IFNγ to level of controls. However, T cell-specific deletion of Notch-pathway components have not shown defects in Th1 differentiation *in vivo* [58, 63–66]. Recent work has found that the association between γ-secretase, Notch and Th1 is complex. In a model of Leishmania infection, T cell-specific deletion of the Notch1 and Notch2 receptors impaired protective Th1 differentiation while deletion of RBP-jk had little effect [66]. Intriguingly, GSI administration in models of allergy-induced asthma were found to inhibit Th2 responses by potentiating Th1 differentiation [22, 67]. We conclude that PSEN1/γ-secretase regulate Th1 differentiation through a T cell-intrinsic mechanism, however, the effects are context-dependent.

### Th17 differentiation is regulated by γ-secretase *in vitro* but not *in vivo*

T cells with a Th17 phenotype are thought to be a critical pathogenic effector cell type in EAE. Th17 differentiation is regulated through activation in the presence of pro-inflammatory cytokines including IL-1, IL-6, IL-23 and TGFβ. Our data demonstrate that γ-secretase interacts with Th17 differentiation *in vivo* and *in vitro*. Treatment with GSI reduced the presence of IL-17 expressing T cells in CNS (Fig 1). During priming, GSI administration reduced the numbers but not the percentages of MOG-specific CD4 T cells expressing IL-17 and RORγt (Fig 2). T cells that lack PSEN1, however, developed normal percentages of Th17 cells following immunization (Fig 6). *In vitro* models demonstrated that disrupting γ-secretase by GSI (Fig 5) significantly inhibited the expression of IL-17, IL12Rb1, IL23R and RORγt. Th17 T cells generated in the presence of GSI were found to have reduced pathogenic function. In fact, GSI and PSEN1 cKO T (Fig 7) cells showed reduced IL-17 and RORγt expression to all tested Th17 polarizing conditions. This suggests that γ-secretase-dependent signals promote Th17 differentiation in a T cell-intrinsic manner; yet, defects in γ-secretase can be overcome during inflammation. It is intriguing to speculate that γ-secretase might regulate Th17 differentiation and survival through a cytokine regulator such as IL-21. IL-21 functions an autocrine regulator to promote Th17 differentiation and survival [68, 69]. In the context of inflammation, the pro-Th17 functions of IL-21 might be compensated by IL-6 or IL-23[70, 71].

Notch signals have been associated with Th17 differentiation *in vitro* [32, 72, 73] and *in vivo* [73–75]. In EAE, the T cell-specific loss of Notch signals did not inhibit the initial generation of IL-17 expressing T cells from naïve precursors but reduced their pathogenicity by regulating the expression of IL-23R or alterations in CNS migration [48, 53]. Additionally, manipulating Notch ligands were found to alter Th17 responses in EAE [74]. The effects of GSI and PSEN1 cKO did not recapitulate the phenotypes described in T cell-specific Notch mutants. This highlights the underlying complexity of Th17 differentiation and the roles of γ-secretase and Notch in this process.

In summary, GSI treatment impacted the generation of autoimmune effector T cell responses *in vivo* by influencing T cell proliferation, Th1 and Th17 differentiation, but these effects were not T cell-intrinsic. GSI treatment did not impact the frequency of T cells expressing GM-CSF suggesting a mechanism to explain the lack of clinical efficacy of GSI treatment [7, 13, 14, 76, 77]. Ultimately, we conclude that PSEN1 and γ-secretase are not essential for MOG35-55-induced EAE. The apparent disconnect between the *in vitro* and *in vivo* effects of disrupting PSEN1 and γ-secretase support the need for stringent *in vivo* models as we seek to unravel the immunology of complex diseases such as multiple sclerosis.

## Supporting information

S1 Arrive Checklist (PDF)
